# Early identification and delay to treatment in myocardial infarction and stroke: differences and similarities

**DOI:** 10.1186/1757-7241-18-48

**Published:** 2010-09-06

**Authors:** Johan Herlitz, Birgitta WireklintSundström, Angela Bång, Annika Berglund, Leif Svensson, Christian Blomstrand

**Affiliations:** 1 Institute of Medicine, Department of Molecular and Clinical Medicine, Sahlgrenska University Hospital, SE-413 45 Göteborg, Sweden; 2 School of Health Sciences, University of Borås, the Pre-hospital Research Centre in Western Sweden, SE-501 90 Borås, Sweden; 3 Stockholm Pre-hospital Centre, South Hospital, SE-118 83 Stockholm, Sweden; 4 Institute of Neuroscience and Physiology, Department of Clinical Neuroscience and Rehabilitation. Sahlgrenska Academy at Gothenburg University, Guldhedsgatan 19, SE-413 45 Göteborg, Sweden

## Abstract

**Background:**

The two major complications of atherosclerosis are acute myocardial infarction (AMI) and acute ischemic stroke. Both are life-threatening conditions characterised by the abrupt cessation of blood flow to respective organs, resulting in an infarction. Depending on the extent of the infarction, loss of organ function varies considerably.

In both conditions, it is possible to limit the extent of infarction with early intervention. In both conditions, minutes count.

This article aims to describe differences and similarities with regard to the way patients, bystanders and health care providers act in the acute phase of the two diseases with the emphasis on the pre-hospital phase.

**Method:**

A literature search was performed on the PubMed, Embase (Ovid SP) and Cochrane Library databases.

**Results:**

In both conditions, symptoms vary considerably. Patients appear to suspect AMI more frequently than stroke and, in the former, there is a gender gap (men suspect AMI more frequently than women).

With regard to detection of  AMI  and stroke at dispatch centre and in Emergency Medical Service (EMS) there is room for improvement in both conditions. The use of EMS appears to be higher in stroke but the overall delay to hospital admission is shorter in AMI. In both conditions, the fast track concept has been shown to influence the delay to treatment considerably.

In terms of diagnostic evaluation by the EMS, more supported instruments are available in AMI than in stroke. Knowledge of the importance of early treatment has been reported to influence delays in both AMI and stroke.

**Conclusion:**

Both in AMI and stroke minutes count and therefore the fast track concept has been introduced. Time to treatment still appears to be longer in stroke than in AMI. In the future improvement in the early detection as well as further shortening to start of treatment will be in focus in both conditions. A collaboration between cardiologists and neurologists and also between pre-hospital and in-hospital care might be fruitful.

## Background

Closer collaboration between disciplines handling various life-threatening complications of atherosclerosis has the potential to improve our understanding of ways of improving treatment. The literature about the early treatment of stroke has mainly appeared during the last decade, whereas similar literature about the heart often appeared 10 years earlier.

One explanation for this difference might be the multidisciplinary nature of stroke management that may include not only emergency physicians but also geriatrics, neurologists, and radiologists most of whom previously had no experience of emergency work. This must have been one important bar to progress.

It is now almost 40 years since the opportunity was first reported, in dogs, to influence the extent of myocardial damage by early intervention with various medications after a coronary artery occlusion [1]. A few years later, further animal experiments indicated that the duration of a coronary occlusion was directly related to the extent of myocardial damage [2]. These research findings form the background to the dramatic evolution in the early treatment of AMI, where "time is muscle" has become a prestige phrase and the limitation of infarct size is the principal aim.

In 1986, the first large-scale study showing a reduction in mortality in men and women with a threatened myocardial infarction, if an intravenous lytic agent was given at an early stage, was published [3]. If this treatment was given within the first hour after the onset of symptoms, the mortality was reduced by 50% [3]. This resulted in the formulation of "the golden hour", which meant that, if patients were treated with thrombolysis within the first hour after the onset of symptoms, 80 lives/1,000 treated could be saved instead of the overall 19/1,000 treated [4]. However, it was also shown that patients with ST-segment elevation myocardial infarction (STEMI) was a subgroup in which very early revascularisation worked [4].

Early trials in stroke in the pre-computed tomography (CT) era in the 1960 s and 1970 s were discouraging due to haemorrhages and increased mortality. In the post-CT era in the 1980 s, increasing optimism was still hampered by the early negative results.

In 1995, it was first reported from a randomised study that treatment with an intravenous lytic agent in acute ischemic stroke resulted in an improved prognosis [5]. These findings of a similar positive effect by a lytic agent in the setting of an acute infarction in two different organs (the heart and the brain) call for an evaluation of the differences and similarities between AMI and acute ischemic stroke. A comparison of this kind will include various aspects of the early phase, including patient factors, community factors and health care system factors. In one previous single county report EMS response times did not differ between AMI and stroke patients [6].

## Differences in pathophysiology explaining differences in possibility for early intervention

In stroke, the mechanism behind symptom onset can be either an infarction through different mechanisms such as lacunar stroke, cerebral embolus, arteriosclerotic large vessel disease or a haemorrhage [7], whereas, in AMI, a fresh occluding or non-occluding thrombus is usually the cause of the symptoms, although other mechanisms such as hypotension or spasm are a possibility [8]. It is estimated that about 10% of all strokes are caused by a haemorrhage. On the other hand, many patients with a fresh occluding thrombus in a coronary artery die suddenly and are therefore not available for treatment. Sudden death due to stroke is more commonly the result of a haemorrhagic stroke, particularly subarachnoid haemorrhage.

In a comparison between stroke and AMI, it seems appropriate, if possible, to restrict the comparison to cases in which an ischemic event is the cause of organ damage. In AMI we aimed, when possible, to focus on STEMI.

## Methods

In February and June 2010, literature searches were performed in the PubMed, EMBASE (Ovid SP) and Cochrane Library databases. Variation of the following terms were used, adopted for each database:

Databases using the following terms:

(Acute myocardial infarction OR AMI OR acute coronary syndrome OR ACS) AND hospital arrival OR arrival times OR delay OR delays AND (Ambulance OR ambulances OR emergency service). In the search for stroke the word stroke replaced acute myocardial infarction OR AMI OR acute coronary syndrome OR ACS. A decision was also made to limit all searches to articles published in English only.

An example of number of hits is shown below for Embase and 'acute myocardial infarction'.

### Search history (159 hits)

#### EMBASE

1. acute myocardial infarction.mp. or exp acute heart infarction/(44307)

2. ami.mp. (9661)

3. acute coronary syndrome. mp. or exp acute coronary syndrome/(10794)

4. acs.mp. (5890)

5. 1 or 2 or 3 or 4 (57652)

6. hospital arrival.mp. (301)

7. arrival times.mp. (257)

8. delay.mp. (79237)

9. delays.mp. (19917)

10. 6 or 7 or 8 or 9 (94116)

11. ambulance.mp. or exp ambulance/(4499)

12. ambulances.mp. (546)

13. emergency service.mp. or exp emergency health service/(16477)

14. 11 or 12 or 13 (19757)

15. 5 and 10 and 14 (191)

16. limit 15 to english language (159)

In all, 433 articles were found for AMI and 186 for stroke.

In all, there are 226 references in this article. However, some of them have been obtained from other sources (mainly from various experts in the field. Some were found in the reference lists from the articles found in the search. Furthermore, some of the references cannot be found from our search words but are still relevant for the completeness of this article.

Sixty-six of the articles found for AMI are referred to in the article. The corresponding figures for stroke is 58.

## The Review

### Delay

#### Various components of delay

In both AMI and stroke, the delay can be divided into various components, where the pre-hospital and in-hospital delays make up the two main components.

In terms of both AMI and stroke, the patient's decision time accounts for the largest part of the pre-hospital delay [9,10]. The median patient decision time has been reported to be fairly similar in AMI (60 min) [9] and stroke (60 min-90 min) [10-12]. However, research on patients' decision time is limited and most research has focused on the delay between the onset of symptoms and admission to hospital, i.e. the total pre-hospital delay. This is associated with some problems, as, with regard to AMI in particular, the EMS systems have become more active on the scene (giving the patient various medications) over the years, thereby prolonging the delay between the onset of symptoms and admission to hospital. The patient decision time, on the other hand, is sometimes difficult to determine. In all probability, the best determinant of the patient decision time is the time between the onset of symptoms and the time of calling for EMS.

With regard to the in-hospital delay, AMI and stroke involve different parameters.

In AMI, the critical time is the time between admission to hospital and the time of admission to the catheterisation laboratory.

In acute stroke, the critical time is divided into two parts:

1/The time between arriving at hospital and CT scan and 2/The time between CT scan and the start of fibrinolysis. Further, an important time point is the time between stroke onset and care in a comprehensive stroke unit.

In terms of both AMI [13-15] and stroke [16-19], it has been clearly shown that the activation of the EMS system can function as a facilitator for shortening the in-hospital delay (including time to CT as well as time to treatment with fibrinolysis and percutaneous coronary intervention (PCI)).

In AMI, the introduction of the pre-hospital ECG has led to improved triage in the field [20-23], resulting in a more rapid preliminary diagnosis, the possibility to start early reperfusion therapy on scene, and finally in the possibility to prepare the hospital for a direct transport to catheterization laboratory and early PCI. A similar instrument has not yet been proven in stroke.

#### Changes in delay

Despite large-scale efforts to reduce the delay between the onset of symptoms and the patient's decision time and admission to hospital respectively, the results have not been particularly impressive. In Sweden, the pre-hospital delay in AMI has not changed much during the last 10 years [24], but this should be related to the opportunity to increase the on-scene time for the EMS system. Nor has there been any marked decrease in the in-hospital delay [24].

In 4 USA communities there was no change in prehospital delay time between 1987 and 2000 [25]

However, among patients with STEMI the fast track concept appear to have reduced delay times (see separate chapter). Among stroke patients, there have been only small changes in delay during the last two decades [26-29], but some changes were reported in Afro-Americans [29]. In 1993/1994, 17% of Afro-Americans arrived at hospital within three hours as compared with 26% in 1999. However, during the last few years, an increased focus on early diagnosis and rapid delivery has hopefully changed the situation [30,31].

#### Variability in delay

There is large variability in the delay, caused among many things by geographical and cultural factors. In AMI, the delay from the onset of symptoms to admission to hospital has varied between regions [15,32-46]; (Table 1). Both STEMI and non-STEMI were included. However, STEMI is associated with a shorter prehospital delay [47,48]. Also in stroke, the delay between onset of symptoms and hospital admission has been reported to vary markedly between different geographical regions [10,12,49-79]; (Table 2). These studies were performed between 1993 and 2009. In the majority of studies, strokes of all types were involved and both women and men were included.

**Table 1:** Delay from symptom onset to arrival in hospital in AMI (including studies published the year 2000 and later)

Ref	Diagnosis	n	Country	Delay (hour; median)	Year
38	AMI	526	Italy	3.5	2001

39	AMI				2003

		192	USA	3.5	

		127	South Korea	4.5	

		136	Japan	4.5	

		141	England	2.5	

		317	Australia	6.5	

41	AMI	194	USA	3.0	2003

40	ACS	250	Denmark	2.0	2004

42	ACS	100	New Zealand	4.0	2006

43	AMI	239	USA	2.5	2006

44	AMI	178	Turkey	2.0	2006

45	ACS	204	Lebanon	4.5	2006

46	ACS	1939	Sweden	2.5	2007

**Range: **2.0 h-6.5 h Mean = 3.5 h

**AMI **= Acute myocardial infarction

**ACS **= Acute coronary syndrome

**Table 2:** Delay from symptom onset to arrival in hospital in stroke including studies (published the year 2000 and later)

Ref	Diagnosis	N	Country	Delay (hour; median)	Year
58	Stroke	1207	USA	2.5	2000

59	Stroke	739	United Kingdom	6.0	2002

60	Stroke	16.922	Japan	6.0	2004

61	Stroke	558	Germany	2.5	2004

62	Stroke	100	Greece	3.0	2004

10	Stroke	196	Taiwan	5.5	2004

12	Stroke	229	Turkey	1.5	2005

64	Stroke	423	Spain	4.0	2005

50	Stroke	130	Japan	7.5	2006

66	Stroke or TIA	615	Switzerland	3.0	2006

68	Stroke	209	Israel	4.0	2006

69	Stroke	150	Australia	4.5	2006

70	Stroke	7901	USA	2.0	2007

72	Ischemic stroke	256	Korea	13.0	2007

73	Ischemic stroke	100	Singapore	16.0	2007

74	Ischemic stroke	129	Taiwan	1.0	2007

78	Stroke	400	USA	3.5	2008

77	Stroke	165	Pakistan	6.0	2008

76	Stroke	375	Italy	5.5	2008

79	Stroke	331	Switzerland	3.5	2009

**Range: **1.0 h - 16.0 h. Mean = 5 h

**TIA = **Transitory Ischemic Attack

In stroke, it has also been shown that various aspects of delay were prolonged during the night [49,52,64,67]. Specific hours during the night for increased risk were not defined. Similar findings were sporadically reported in AMI [80]. In stroke, a shorter delay has been reported at the weekend [81,82]. In one study, 27% of patients arrived at hospital within one hour after symptom onset on Sundays compared with only 11% on other days of the week [82].

#### Delay and gender

In AMI, the results have consistently (in Sweden, the USA, the Netherlands and France) indicated that women have a prolonged pre-hospital delay, as compared with men, which is caused by a prolonged decision time [83-87], but doctors' delay might also contribute [88]. The delay between onset of pain and arrival at hospital in these studies was around 30 minutes longer in women than in men.

Although a few reports have indicated similar findings in stroke [16,68,69,16,89], the overall results have not been as consistent and the opposite findings [90] or no difference [48,71,91] have also been reported.

There is still insufficient knowledge about the way women make decisions in the early phase of AMI and stroke [92,93]. Even the in-hospital delay in AMI has been reported to be prolonged in women [87,94-96] and similar results were found in some stroke surveys [16,58]] [97,98] but not in others [30,56,59,99].

#### Delay and previous cardiovascular disease

In overall terms, it does not appear that a history of previous infarction [100] or stroke has a major impact on delay in either AMI or stroke.

With regard to AMI, the reports on the influence of previous infarction on delay have been inconsistent; some suggest that a previous history of infarction reduces delay [85,101], while this was not confirmed by others [102]. Even the opposite has been reported [80]. A history of hypertension [36], as well as diabetes [33-35,37], has been shown to be associated with a prolonged pre-hospital delay. Furthermore, the presence of pre-infarction angina has been associated with a longer pre-hospital delay [103].

With regard to stroke, it has been reported that a previous stroke is associated with a shorter delay [104], but the opposite has also been found [66]. It has been suggested that a previous coronary event shortens the delay in acute stroke [53], whereas a history of diabetes appears to be associated with a prolonged delay [67,81].

#### Ethnicity and delay

An increased pre-hospital delay in AMI was found among the Asian and Latino population in the USA [105]. In the USA, door-to-ECG time at the emergency department was longer in non-white populations [106]. In the United Kingdom, South Asians used EMS less frequently in acute chest pain [107].

Afro-Americans had a longer delay in stroke [108]. However, among stroke patients the delay from 911 call to arrival in Emergency Department (ED) was not substantially influenced by living in poorer areas or ethnicity [109].

### Patient factors

#### Symptom onset

There are occasional difficulties defining symptom onset in AMI as well as stroke. In AMI, there is sometimes a stuttering start of the pain, in both women and men, where there are difficulties delineating the true onset of infarction [103]. In stroke, some patients wake up with hemiparesis or dysarthria [70]. In both conditions, it is difficult to estimate how often there are uncertainties in the estimation of onset of infarction. However, a number of stroke surveys have estimated this figure, ranging from 19%-35% [61,65,110,111]. These surveys were performed in the latter part of the 1990 s and the beginning of the 21st century, including both men and women and both ischemic and haemorrhagic stroke. The largest of these cohorts comprised 2,165 patients. In stroke, a transient ischemic attack (TIA) and in AMI, unstable angina can precede the major episode, making it difficult to establish the exact onset time.

#### Type of symptom

In AMI, a variety of symptoms have been described. The classical type, with the acute onset of severe pain associated with a cold sweat, has been reported to occur in about 20% of AMI cases in both women and men [112], however more frequently in STEMI. Other types include the more gradual onset of pain or pain that comes and goes [113]. However, a large number of patients have symptoms other than chest pain [114].

In stroke, there are a number of types of symptom onset, with sudden onset such as hemiparesis, hemihypesthesia, loss of vision in one or both eyes, speech problems, loss of consciousness, sudden headache, dizziness and balance problems [81]. Also in stroke, there is sometimes a more gradual increase in symptom severity and there are sometimes atypical symptoms as well [115].

#### Symptoms and delay

A sudden onset of symptoms in AMI has been reported to be associated with a shortened delay to hospital admission [48]. Conscious disturbances have been shown to shorten delay in stroke [116]. Similarly, more severe strokes have been associated with a shorter delay [81][108][117]. The increasing delay in women in AMI but also in some studies of stroke has been explained by more atypical symptoms in women [115].

Loss of consciousness and difficulty speaking shortened door-to-doctor time and door-to-image time in stroke [98].

#### Recognition

In AMI, with few exceptions, about 75% suspect a heart attack [46,118,119], more frequently in men than women [46]. The relationship between expected and perceived symptoms appears to be important for the decision process in AMI [120,121]. Symptom recognition is an important factor in reducing delay in AMI [40,122].

In stroke, about 25% - 50% of patients suspect stroke [11,67,78,123-126]. Awareness of stroke has been associated with a shorter delay [79,127].

#### The use of the EMS

In AMI, the use of the EMS may vary between continents. In Europe and Australia, the figures often reach more than 50% [80,128-130], whereas in the United States the figures are often around 50% or lower [131-133]. In 4 USA communities the use of EMS increased from 37% in 1987 to 44% in 2000 (p < 0.0001) [25]. In China and Singapore, clearly less than 50% of AMI patients used EMS [134,135].

Patients with STEMI more frequently use an EMS [136]. More rapid definitive care is usually obtained by using the EMS [130,133,135,137].

In stroke, the use of the EMS varies markedly between 12% and 69% [30,62,66,16,117,59,60,125,138-142]. These surveys mostly include stroke in general, but some only include ischemic stroke.

Factors associated with the use of the EMS in AMI have been reported to be

1) Knowledge of the importance of quickly seeking medical care,

2) Abrupt onset of pain reaching maximum intensity within minutes,

3) Nausea or cold sweat,

4) Vertigo or near syncope,

5) ST-elevation ACS,

6) Increasing age,

7) Previous history of heart failure,

8) Long distance to hospital [136].

In stroke, these factors were older age and when someone other than the patient identified the problem [143].

The use of the EMS in stroke has been associated with a shorter delay to hospital admission and, furthermore, to a shorter in-hospital delay to treatment [16,17,59,69,76,79,90,123,144-146].

Survival in relation to the use of EMS has not been clearly addressed, most probably because patients who use the EMS are older and have a different co-morbidity.

### Community factors

#### Knowledge

In a 1995 survey in USA 89% of adult respondents reported the warning signs of a heart attack correctly [147].

The knowledge of stroke has been evaluated in a number of community surveys. A surprisingly high percentage (about 50%) do not recognise the most typical warning signs of stroke [148-152].

In one survey in Spain, 60% were unable to describe any warning signs for stroke [153]. Similar observations were made among uninsured Latino immigrants in the USA [154].

#### Knowledge and delay

The knowledge of the importance of quickly seeking medical assistance shortens the pre-hospital delay and increases the use of the EMS in AMI [136,155,156]. Physicians have a shorter pre-hospital delay in AMI [156,157]. In many patients, a heart attack differs considerably from their concept of a heart attack [121,158,159]. Mismatch has been reported to be as high as 58% [121].

Similar findings have been reported in stroke [30,77,160]. However, in one survey, knowledge of stroke was not associated with delay [161].

#### Intervention to improve knowledge

In the 1980 s and 1990 s, educational campaigns were started to increase the use of the EMS and reduce prehospital delays in AMI. In Europe, some reduced delays [155,156] and, in the USA, some increased the use of the EMS [132]. However, overall these campaigns did not markedly change the situation [162,163].

An educational campaign in Carolina increased the percentage of stroke patients who reached hospital within 24 hours [164]. Similar experiences were reported by others [165]. A population based stroke intervention trial in Berlin was effective in reducing prehospital delay in women but not in men [166].

In Texas, one educational intervention increased the percentage of stroke patients who received thrombolysis [167] and another potentially improved the intention to call 911 for stroke among school children [168].

Public education has increased the proportion of inhabitants who can identify stroke warning signs [169].

#### Witness and delay

In both AMI and stroke, the importance of relatives, friends or others with regard to delay has been highlighted [11,116,170]. This is particularly relevant in stroke, due to a more common patient incapacity.

Both patients who have suffered an AMI and their relatives appeared to act more appropriate to someone else's chest pain than to their own [171]. Patients with AMI often seek advice from family members and friends at symptom onset [9,46,170,172-176] and significant others appear to play a vital role in shortening the patient's decision time process in AMI [172], simply because of patient denial. Patients view of trustworthiness of others also seem to influence delay in AMI [177].

### System factors

#### Recognition at dispatch centre

The opportunity for the early identification of AMI at the dispatch centre has been evaluated [178,179]. Although the experiences were relatively positive, it has been suggested that computer algorithm support might increase the diagnostic accuracy still further [180].

In stroke about 30% were identified by dispatchers [142,181].

#### Recognition by EMS

Early identification of AMI by the rescue team on the scene has been evaluated [182,183]. The pre-hospital ECG has markedly improved the diagnostic accuracy, particularly with regard to STEMI [184].

Analysis of biochemical markers has not improved the diagnostic accuracy in a similar manner [185]. Therefor the preliminary diagnosis of AMI or ACS on the scene is currently based on clinical history, clinical examination and ECG.

In stroke, the rescue team has to rely on clinical history and clinical examination. Diagnostic scales to identify stroke patients have also been used [186]. With the support of the Face, Arm, Speech test, stroke diagnosis by paramedics has been reported to be 79% [187]. The diagnostic accuracy of stroke in the pre-hospital setting has not yet been evaluated as extensively as that of AMI, but one study found that thrombolytic checklists to identify eligible stroke patients are used more frequently (37%) than checklists to identify eligible AMI patients (28%) [186].

#### Pre-hospital treatment

In AMI, various therapeutic alternatives, including thrombolysis [188], nitroglycerine [189], aspirin [190] and beta-blockers [191], have been introduced in clinical routines.

In stroke, pre-hospital neuroprotective therapy has been started at research level [192].

#### Fast track

A number of studies have highlighted the value of fast-tracking patients with STEMI directly to the coronary care unit or catheterisation laboratory. This has been shown not only to shorten delay to treatment but also to improve outcome [193-202]. The training of the staff in hospital and the implementation of guidelines or an audit programme can also reduce the in-hospital delay to treatment in AMI [203-205].

It was recently shown that half of AMI patients admitted to the ED were given inappropriately low levels of triage [206]. The median door-to-ECG time was 12 min and the median door-to-thrombolysis time was 40 min [206]. One disturbing factor is the level of crowding at the ED [207].

A prolonged door-to-ECG time at the ED was associated with a poorer outcome [208]. The door-to-ECG time can be reduced by implementing a triage process [209].

Similar experiences were found in stroke [210-212]. It was shown that a rapid response system in hospital could reduce the delay in stroke [213].

A pre-hospital notification increased the use of thrombolysis from 6% to 14% [214]. Similarly, an acute stroke team at the ED increased the use of thrombolysis [215].

A pre-hospital acute stroke triage protocol has also been shown to reduce the pre-hospital and in-hospital delay [216]. A Computerised Physician Order Entry-Based Stroke team approach programme significantly reduced the time from ED arrival to evaluation and treatment [217]. However, another approach in stroke is to introduce CT scanning at the ED [31]. This increased the eligibility for thrombolysis in stroke dramatically [31]. A fast track based on a competent pre-hospital clinical evaluation can also directly transfer patients to the stroke unit via the CT scanner.

A multilevel educational programme improved rapid hospitalisation and paramedic diagnostic accuracy in acute stroke, which also increased the number of patients within the three-hour tissue plasminogen activator window [30].

### Final comments

Acute myocardial infarction and acute ischemic stroke are two conditions which are suitable for early revascularisation, which improves outcome. The delay from symptom onset to the delivery of treatment is therefore of the utmost importance in both conditions. In stroke, there is a drawback, as a possible haemorrhage must be excluded before thrombolysis can be delivered. In AMI, only patients with STEMI (about one third of all patients with AMI) have been shown to benefit from very early revascularisation, today usually PCI.

Telemedicine can help collaboration between smaller community hospitals and the large central hospital both in AMI [20,218-220] and in stroke [221-225]. Telephone guidance of systemic thrombolysis in acute ischemic stroke is another approach [226].

The goal of more rapid delivery of treatment could perhaps be achieved by a reduction in patient decision time, an improvement in early identification and an improvement in logistics, including fast tracking (transporting the patient directly to the catheterisation laboratory in AMI and to the CT scan and stroke unit in stroke). Bridging therapies with intra-arterial thrombolysis or thrombectomy calls for the perfection of logistics, pre-hospital care and collaboration between hospitals.

Efforts to improve the early chain of care in AMI have been ongoing for the last two decades. Similar efforts in acute ischemic stroke have been in progress for the last decade.

In all probability, representatives from these two disciplines (cardiology and neurology) can learn from one another, with the common goal of limiting organ damage and thereby improving outcome in terms of both mortality and morbidity.

## Competing interests

The authors declare that they have no competing interests.

## Authors contributions

JH is responsible for the design of the manuscript, the literature search and the writing of the manuscript. BW has contributed with constructive comments and references. ABe has contributed with valuable background information which was of importance for the design and content of the manuscript. ABå has contributed with valuable background information which was of importance for the design and content of the manuscript

LS has contributed with valuable background information which was of importance for the design and content of the manuscript. CB has contributed with constructive comments and references

All authors have read and improved the final manuscript
